# Dietary Vitamin B_6_ Intake Associated with a Decreased Risk of Cardiovascular Disease: A Prospective Cohort Study

**DOI:** 10.3390/nu11071484

**Published:** 2019-06-29

**Authors:** Jimin Jeon, Kyong Park

**Affiliations:** Department of Food and Nutrition, Yeungnam University, 280 Daehak-ro, Gyeognsan, Gyeongbuk 38541, Korea

**Keywords:** cardiovascular disease, vitamin B_6_, men, cohort study

## Abstract

Although the biological mechanisms underlying the beneficial effects of vitamin B_6_ on cardiovascular disease (CVD) have been reported on, epidemiological studies have yielded controversial results, and data on the Korean population are limited. This study examined the association between dietary vitamin B_6_ intake and CVD incidence in Koreans. A total of 9142 participants of the Korean Genome and Epidemiology Study, aged 40–69 years, who did not have CVD or cancer at the baseline were included in the analysis. Dietary data were assessed using a validated semi-quantitative food frequency questionnaire. CVD incidence was assessed using biennial questionnaires and confirmed through repeated personal interviews. Multivariable-adjusted hazard ratios (HRs) and 95% confidence intervals (CIs) were estimated using Cox proportional hazard regression models. After multivariate adjustment, a higher vitamin B_6_ intake was significantly associated with a decreased CVD risk in men (HR: 0.44; 95% CI: 0.25–0.78); no such association was observed in women. Dose-response analysis confirmed the presence of inverse linearity between vitamin B_6_ intake and CVD incidence in men (*p* for nonlinearity = 0.3). A higher dietary intake level of vitamin B_6_ was associated with a reduced CVD risk in Korean men. These observations require further verification in other populations.

## 1. Introduction

Cardiovascular disease (CVD) is responsible for a third of all global deaths [1], and the prevalence of CVD is increasing worldwide [2] as well as in Korea [3]. As the rise in the CVD incidence poses an economic burden and leads to increases in the number of disability-adjusted life years, indicating a diminished quality of life, it is important to identify and understand the potential risk factors and protective factors that may alleviate the burden of this disease [4].

Certain nutrients have long been highlighted as modifiable risk factors for CVD [5,6]. Previous studies have investigated the possible biological mechanisms underlying the beneficial effects of vitamin B_6_ on CVD prevention, such as the inhibition of lipoperoxide production and decreases in the levels of homocysteine and inflammation which are known risk factors of CVD [7]. In addition, vitamin B_6_ deficiency can cause hyperhomocysteinemia [8], which may lead to arterial wall damage [7]. Nevertheless, epidemiological studies investigating the association between vitamin B_6_ and CVD have shown inconsistent results [9,10,11]. The Nurses’ Health Study in the United States demonstrated an inverse relationship between plasma vitamin B_6_ levels and the risk of myocardial infarction [9]. Similarly, a Japanese cohort study revealed an inverse association between vitamin B_6_ intake and coronary heart disease (CHD) incidence [10]. In contrast, a study conducted in Finnish men found no significant association between vitamin B_6_ intake and CVD risk [11]. In Korea, few studies have investigated the effect of vitamin B_6_ intake on CVD, and no corresponding prospective analyses have been conducted to date. Two previous case-control studies analyzed the association between vitamin B_6_ and stroke in the Korean population and yielded conflicting results [12,13]. Those case-control studies were limited by their design, which aggravated the analysis of long-term dietary effects on chronic diseases, as it is practically impossible to recall long-term dietary habits prior to disease diagnosis [14,15]. 

Further investigation of the longitudinal association between vitamin B_6_ and CVD is warranted in a prospective cohort study, as is the assessment of dietary intake prior to disease onset. We, therefore, prospectively examined this association among Korean adults enrolled in the Ansung–Ansan cohort study.

## 2. Materials and Methods 

### 2.1. Study Population

The present study analyzed data obtained in one of the prospective cohort studies that were part of the Korean Genome and Epidemiology Study. The baseline survey of the community-based Ansung–Ansan cohort study was conducted between 2001 and 2002 in 10,030 Korean adults aged 40–69 years who were residing in the Ansung (rural) and Ansan (urban) areas of Gyeonggi province. Participants were randomly recruited from enrolled inhabitants for statistically reliable results, and the general characteristics of participants from both areas were similar to those who were not recruited (Ansung, *n* = 5018, response rate = 69.6%; Ansan, *n* = 5012, response rate = 45.7%). The follow-up examination was conducted every two years thereafter. Details on the baseline recruitment and follow-up survey have been presented elsewhere [16]. During each examination, the participants’ demographics, lifestyle, environmental factors, family history of disease, medical history, and diet were assessed with a structured questionnaire. Data collection was performed by trained investigators according to standardized procedures. We used baseline data and data obtained during follow-ups that were conducted until 2012 in this study.

Exclusion criteria were having a history of CVD or cancer, taking medications, or receiving treatments for CVD-related diseases (*n* = 498) at the baseline. Participants with no available data on vitamin B_6_ consumption (*n* = 307) or with an implausible daily energy intake level <500 kcal or >5000 kcal (*n* = 83) were also excluded [17]. A total of 9142 participants were finally included in the study (Figure S1). 

Informed consent was obtained from all participants and the study was approved by the Korea Centers for Disease Control and Prevention Institutional Review Board (IRB number: KU-IRB-15-EX-256-A-1) and the Yeungnam University Institutional Review Board (IRB number: 7002016-E-2016-003).

### 2.2. General Characteristics and Anthropometric Measurements

The participants’ demographic data (sex, age, residential area, educational levels, monthly household income) and lifestyle data (smoking status, alcohol consumption, physical activity levels) were collected using questionnaires. The monthly household income was classified into four groups, ranging from <1,000,000 Korean Republic Won (KRW) to ≥4,000,000 KRW. The educational levels were categorized into: elementary school graduate or lower, middle school graduate, high school graduate, and college graduate or higher. By smoking status, participants were categorized into the current smoker and non-smoker groups. For alcohol consumption, we used participants’ weekly intake frequencies (glasses/week) and grouped them into the non-drinker and drinker groups. Physical activity levels were quantified using the daily exercise hours and intensity of physical activities (light, moderate, and vigorous) based on metabolic equivalents [18]. Anthropometric measurements such as height and weight were conducted by trained technicians. Body mass index (BMI) was calculated by dividing the body weight (kg) by the height squared (m^2^). 

### 2.3. Dietary Assessment

Dietary information was assessed during the baseline survey (2001–2002) and the second follow-up survey (2005–2006) using a semi-quantitative food frequency questionnaire (SQFFQ), the validity and reproducibility of which have been proven [19]. In brief, the frequency of intake was assessed by offering nine possible responses for each food, ranging from “almost never” to “3 times per day”. The portion size of each food could be estimated as “0.5 times the reference”, “reference”, or “1.5–2 times the reference”. The food consumption levels were determined by weighing the portion size and multiplying it with the weekly frequency of each food (servings per week). Daily nutrient and total energy intake values were calculated based on dietary information and the food composition database of the Rural Development Administration of Korea [20]. The mean dietary information values from the two surveys were used to minimize misclassification errors. Fully conditional specification imputation procedures were used to handle missing values of dietary data in the second follow-up survey. We imputed missing values of second follow-up dietary data from their posterior predictive distribution using regression equations with baseline data [21]. Data on the use of dietary supplements (yes/no) were obtained from a subset of the dietary questionnaire.

### 2.4. Ascertainment of CVD

CVD-related data were obtained through biennial self-reported questionnaires and confirmed by trained investigators during individual participant interviews. CVD incidence was established if participants had been newly diagnosed or were taking medications or receiving treatments for one or more of the following diseases: stroke, coronary artery disease, myocardial infarction, and/or cerebrovascular disease.

### 2.5. Statistical Analysis

The person-years of each participant were computed as the period between the date of baseline registration and study end date, which was defined as the date of the first CVD diagnosis or last known alive for participants without CVD. Study participants were divided into quintiles based on their dietary intake levels of vitamin B_6_. Continuous and categorical variables were analyzed for each quintile using a general linear model or chi-square test, respectively. All nutrient intakes were adjusted for total energy intake using the residual method [17]. Nutrient patterns were derived from those of 20 nutrients (without vitamin B_6_) through principal component analysis (PCA) [22], performed using varimax rotation with 20 nutrients. Major nutrient patterns were extracted based on a scree plot as well as eigenvalues >1.0. The factor loading matrix of nutrients is presented in Table S1. In total, three nutrient factors were identified: Factor 1, with high loading for fiber, folate, carotene, vitamin A, potassium, vitamin C, iron, sodium, vitamin E, calcium, and phosphorus; Factor 2, with high loading for carbohydrate, protein, fat, thiamin, and niacin; and Factor 3, with high loading for retinol, cholesterol, calcium, riboflavin, and phosphorus. Cox proportional hazard regression models were employed to estimate hazard ratios (HRs) and their confidence intervals (CIs) for the risk of CVD according to dietary intake levels of vitamin B_6_. Possible effect modifiers were determined for demographic variables, lifestyle, and history of disease using both linear and Cox proportional hazard regression models. Multiple confounding factors were identified based on preliminary analysis and literature review [23,24,25]. Four covariate models were evaluated: Model 1, unadjusted; Model 2, adjusted for age; Model 3, further adjusted for monthly household income, BMI, educational levels, physical activity levels, residential area, smoking status, alcohol consumption, and hypertension and diabetes (defined as a history of diagnosis or taking medication) at baseline; Model 4, Model 3 with further adjustments for total energy intake, three major nutrient factors identified in the PCA, and use of dietary supplements. The *p* for trend was calculated using the median of the vitamin B_6_ intake quintiles as a continuous variable. Nonlinear relationships were examined using restricted cubic splines. The Statistical Analysis System version 9.4 software (SAS Institute, Cary, NC, USA) was used to perform all analyses and the significance level for all tests was defined as α = 0.05.

## 3. Results

During the mean follow-up period of 7.4 years, 278 and 284 CVD cases were documented among men and women, respectively. The general baseline characteristics of the participants are shown in Table 1. The dietary vitamin B_6_ intake ranges were 1.01–6.22 mg/day in men and 0.88–7.51 mg/day in women, and the average age was 51.47 ± 0.13 years in men and 52.52 ± 0.13 years in women. Almost two-thirds of the alcohol drinkers were male, including 70.78%, 73.52%, and 71.54% of men in the lowest, mid, and highest dietary vitamin B_6_ intake groups, respectively. In contrast, female alcohol drinkers accounted for only one-fourth of all the women, including 21.86%, 25.56%, and 27.57% of those in the lowest, mid, and highest dietary vitamin B_6_ intake groups, respectively. Approximately half the men were current smokers (51.60%, 48.80%, and 50.06% in the lowest, mid, and highest dietary vitamin B_6_ intake groups, respectively.), while the proportion of female smokers was very low (3.62%, 2.45%, and 4.19% in the lowest, mid, and highest dietary vitamin B_6_ intake groups, respectively).

Table 2 shows the HRs of incident CVD in men and women according to the quintiles of dietary vitamin B_6_ intake levels. In the multivariable adjusted model (Model 4), men in the highest quintile of dietary vitamin B_6_ intake were less likely to develop CVD than those in the lowest quintile (HR: 0.44; 95% CI: 0.25–0.78). In contrast, the dietary intake of vitamin B_6_ was not associated with CVD risk in women. 

**Figure 1:**
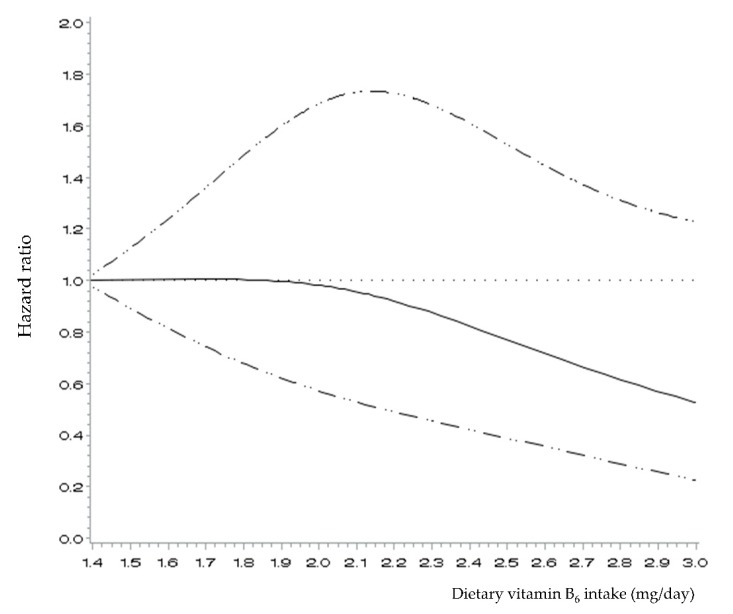
Hazard ratios (95% confidence intervals) for the non-linear relationship between energy-adjusted dietary vitamin B_6_ intake levels and incidence of cardiovascular disease in Korean men, evaluated with restricted cubic splines. The model was adjusted for age, monthly household income, body mass index, educational levels, physical activity levels, residential area, smoking status, alcohol consumption, hypertension and diabetes at baseline, total energy intake, three major nutrient factors identified in the principal component analysis, and use of dietary supplements.

Figure 1 presents the spline curve for the association between dietary vitamin B_6_ intake and CVD incidence among men. An inverse linear association was observed, in that CVD risk decreased with each increment in the dietary levels of vitamin B_6_ intake after about 1.9 mg/day (*p* for nonlinearity = 0.3).

## 4. Discussion

In this prospective study, we evaluated the effect of dietary vitamin B_6_ intake on the risk of CVD among Korean men and women enrolled in the community-based Ansung–Ansan cohort study. As a result, Korean men with a higher dietary intake of vitamin B_6_ had a lower incidence of CVD. This association was not significant in women. Dose-response analyses confirmed the presence of a linear association between higher dietary vitamin B_6_ intake and decreased CVD incidence in men.

Pyridoxal 5′-phosphate (PLP), the active coenzyme form of vitamin B_6_, is required for more than 140 different enzymatic reactions in carbohydrate, amino acid and lipid metabolism, neurotransmitter synthesis, and steroid hormone receptor modulation [26]. PLP is also involved in reactions pertaining to antioxidant activity, the inflammatory process, homocysteine catabolism, and phosphorylation-all metabolic processes that are related to CVD prevention [27]. Oxidative stress, which represents a state of imbalance between oxidants and antioxidants, may occur due to the excessive accumulation of oxidants in the body or due to a lack of antioxidants, and may lead to vascular inflammation and endothelial dysfunction [28]. Homocysteine, an amino acid synthesized during the conversion of methionine to cysteine in one-carbon metabolism [29], is known to increase oxidative stress when deposited in the body [30]. The results of a meta-analysis of prospective cohort studies demonstrated that elevated homocysteine levels are associated with the incidence of CVD, indicating that homocysteine is an independent risk factor for CVD along with oxidative stress [31]. Vitamin B_6_ acts as an antioxidant by scavenging free radicals, reducing lipid peroxidation, and preventing damage to the integrity of the mitochondrial membrane [32]. It is also linked to inflammatory-related functions, which play a key part in the pathogenesis of atherosclerosis [33]. PLP regulates inflammatory reactions through involvement in the regulation of cytokine-induced mechanisms of immunocytes for the activation of the inflammatory reaction as well as synthesis of nucleic acids and proteins [33,34]. Several epidemiological studies have shown that low plasma PLP levels are inversely associated with the major bio-markers of inflammation [33,34,35]. In addition, vitamin B_6_ acts as a cofactor of cystathionine β-synthase and cystathionine γ-cystathionase in the trans-sulfuration of homocysteine to cystathionine and cysteine [36]. A previous study revealed that a higher vitamin B_6_ intake level was associated with a lower plasma total homocysteine level [37]. In other words, vitamin B_6_ may lower the risk of CVD by regulating the homocysteine concentrations in the plasma and indirectly regulating the production of cysteine as a precursor of the antioxidant, glutathione.

Previous epidemiological studies evaluating the association between vitamin B_6_ from diet and plasma PLP levels, and the incidence of CVD yielded inconsistent results [23,24,25,38,39,40]. An inverse association between plasma PLP levels and CHD was previously identified in a cohort study in the United States [40], while a study in the Netherlands reported that plasma PLP concentrations were not associated with CHD risk in women and men [39]. A study in Chinese men demonstrated that a higher vitamin B_6_ intake level was associated with a decreased CVD risk and total mortality [23]. In addition, a study conducted in Finland revealed a significantly decreased risk of stroke in male smokers with a high dietary vitamin B_6_ intake after adjustment for age and dietary supplement intake; however, this association was not significant after adjustment for multiple confounding factors such as alcohol consumption, smoking, and BMI [38]. Furthermore, the Health Professional Follow-up Study found no association between vitamin B_6_ intake and the risk of stroke [25]. A recent meta-analysis demonstrated an inverse association between vitamin B_6_ intake and CHD risk. In addition, a strong inverse association between the intake of vitamin B_6_ and CHD incidence was found in a sensitivity analysis after the exclusion of participants with pre-existing CVD [41]. No such association was observed in women, which is consistent with our results.

In the present study, higher dietary vitamin B_6_ intake levels lowered the CVD risk in men but had no significant effect on the CVD risk in women. This may be attributed to the sex-specific difference in plasma homocysteine levels and the complex interactions between vitamin B_6_, estrogen, and homocysteine. A sex-related comparison in Europe and the United States showed that men had higher levels of plasma homocysteine than women [42,43]. A previous study assessing homocysteine levels in Koreans also demonstrated that the mean homocysteine concentrations and prevalence of hyperhomocysteinemia were significantly higher in men than women [44]. The effects of the female hormone, estrogen, on the cardiovascular system may also mask the health benefits of vitamin B_6_ in women. Estrogen leads to enhanced glutathione levels and prevents peroxynitrite (ONOO-) formation by activating cystathionine β-synthase and regulating enzymes in glutathione synthesis [45]. Furthermore, estrogen is known to prevent the accumulation of low-density lipoproteins in the blood vessel walls, promote the bio-availability of nitric oxide, and improve vascular endothelium function [46]. Nevertheless, the mechanism underlying the sex-dependent effect of vitamin B_6_ on CVD risk is unclear; hence, further research is needed to investigate this association.

Several limitations of this study should be noted. First, the residual confounding effects of unmeasured or unknown variables may have affected the results, although we did adjust for multiple potential confounding factors. Second, we could not obtain data on dietary supplements and plasma homocysteine levels in our study; thus, the interactions between vitamin B_6_ intake (food and supplement), homocysteine levels, and CVD incidence could not be evaluated. Third, the incidence of CVD was assessed based on responses to self-reported questionnaires, which may have led to diagnostic misclassification. However, a previous study reported that the validity of the CVD cases in this cohort was acceptable, showing a 93% concordance between self-reported CVD and medical records. Finally, as the participants of this study were all residents of the Gyeonggi area in South Korea, it may be difficult to generalize the results to other populations. Nevertheless, we aimed to minimize errors in measurement by using the average dietary values of two repeats of a validated SQFFQ. To our knowledge, this is the first study to prospectively evaluate the association between dietary vitamin B_6_ intake and CVD risk in Korean adults.

## 5. Conclusions

In summary, a higher dietary intake level of vitamin B_6_ was associated with a decreased risk of CVD in Korean men but not Korean women. Further large-scale studies need to examine the effect of dietary and supplemental vitamin B_6_ intakes on plasma homocysteine levels in men and women as well as to determine the appropriate intake levels for CVD prevention.

## Figures and Tables

**Table 1:** Baseline characteristics of participants according to sex stratified by energy-adjusted dietary vitamin B_6_ intake levels.

	Men			Women		
	Vitamin B_6_ Intake (Quintile)			Vitamin B_6_ Intake (Quintile)		
	Q1	Q3	Q5	Q1	Q3	Q5
*n*	878	878	878	950	950	950
Range (median), mg/day	1.01–1.83 (1.70)	2.01–2.16 (2.08)	2.40–6.21 (2.62)	0.88–1.73 (1.58)	1.93–2.08 (2.00)	2.30–5.22 (2.49)
Age (years)	54.12 ± 0.29	51.26 ± 0.29	49.86 ± 0.29	55.08 ± 0.29	52.54 ± 0.29	50.23 ± 0.29
Residential area						
Ansung	596 (67.88)	320 (36.45)	329 (37.47)	642 (67.58)	419 (44.11)	495 (52.11)
Ansan	282 (32.12)	558 (63.55)	549 (62.53)	308 (32.42)	531 (55.89)	455 (47.89)
Educational levels						
Elementary school graduation or lower	248 (28.38)	142 (16.21)	130 (14.87)	549 (58.59)	416 (44.11)	316 (33.40)
Middle school graduation	210 (24.03)	188 (21.46)	179 (20.48)	188 (20.06)	213 (22.59)	256 (27.06)
High school graduation	285 (32.61)	346 (39.50)	346 (39.59)	164 (17.50)	249 (26.41)	287 (30.34)
College graduation or higher	131 (14.99)	200 (22.83)	219 (25.06)	36 (3.84)	65 (6.89)	87 (9.20)
Monthly household income (KRW)						
<1,000,000	353 (40.67)	187 (21.37)	169 (19.34)	520 (55.56)	386 (41.15)	301 (32.44)
1,000,000-<2,000,000	269 (30.99)	287 (32.80)	267 (30.55)	217 (23.18)	256 (27.29)	288 (31.03)
2,000,000-<4,000,000	201 (23.16)	302 (34.51)	332 (37.99)	174 (18.59)	237 (25.27)	272 (29.31)
≥4,000,000	45 (5.18)	99 (11.31)	106 (12.13)	25 (2.67)	59 (6.29)	67 (7.22)
Body mass index (kg/m^2^)	23.66 ± 0.10	24.29 ± 0.10	24.79 ± 0.10	24.68 ± 0.11	25.01 ± 0.11	25.00 ± 0.11
Physical activity levels ^a^	25.38 ± 0.58	19.31 ± 0.58	20.31 ± 0.58	22.54 ± 0.53	19.71 ± 0.53	20.23 ± 0.53
Alcohol drinkers	620 (70.78)	644 (73.52)	626 (71.54)	207 (21.86)	241 (25.56)	260 (27.57)
Current smokers	452 (51.60)	428 (48.80)	438 (50.06)	34 (3.62)	23 (2.45)	39 (4.19)
Dietary supplement users	119 (13.55)	130 (14.81)	149 (16.97)	162 (17.05)	236 (24.84)	258 (27.16)
Nutrient factors ^b^						
Factor 1	−0.61 ± 0.03	−0.10 ± 0.03	0.24 ± 0.03	−0.28 ± 0.03	−0.12 ± 0.03	0.73 ± 0.03
Factor 2	−0.20 ± 0.03	0.29 ± 0.03	0.48 ± 0.03	−0.57 ± 0.03	−0.20 ± 0.03	0.17 ± 0.03
Factor 3	−0.22 ± 0.03	−0.14 ± 0.03	−0.22 ± 0.03	0.12 ± 0.03	0.16 ± 0.03	0.22 ± 0.03

Values are mean ± standard error or *n* (%). Q, quintile; KRW, Korean Republic Won. ^a^ Physical activity levels were quantified using the daily exercise hours and intensity of physical activities (light, moderate, and vigorous) based on metabolic equivalents. ^b^ Major nutrient factors identified in the principal component analysis.

**Table 2:** Hazard ratios (95% confidence intervals) for cardiovascular disease risk according to energy-adjusted dietary vitaminB_6_ intake levels stratified by sex.

	Vitamin B_6_ Intake (Quintile)					*p* for Trend
	Q1	Q2	Q3	Q4	Q5	
Men						
*n*	878	879	878	879	878	
Case	72	53	52	65	36	
Person year	7016	6002	5876	6305	7340	
Range (median), mg/day	1.01–1.83 (1.70)	1.84–2.00 (1.93)	2.01–2.16 (2.08)	2.17–2.39 (2.26)	2.40–6.22 (2.61)	
Model 1	1	0.88 (0.62, 1.25)	0.88 (0.62, 1.26)	1.02 (0.73, 1.43)	0.48 (0.32, 0.71)	0.001
Model 2	1	1.04 (0.73, 1.49)	1.11 (0.77, 1.59)	1.42 (1.01, 2.00)	0.68 (0.45, 1.02)	0.3
Model 3	1	0.96 (0.67, 1.39)	0.98 (0.68, 1.43)	1.27 (0.88, 1.82)	0.56 (0.36, 0.86)	0.048
Model 4	1	0.90 (0.62, 1.32)	0.89 (0.60, 1.33)	1.10 (0.72, 1.67)	0.44 (0.25, 0.78)	0.02
Women						
*n*	950	950	950	950	950	
Case	97	53	47	41	46	
Person year	8050	6890	6410	6514	7382	
Range (median), mg/day	0.88–1.73 (1.58)	1.74–1.92 (1.84)	1.93–2.08 (2.00)	2.09–2.29 (2.17)	2.30–7.51 (2.48)	
Model 1	1	0.65 (0.46, 0.90)	0.62 (0.44, 0.88)	0.53 (0.37, 0.77)	0.52 (0.37, 0.74)	<0.001
Model 2	1	0.70 (0.50, 0.98)	0.74 (0.52, 1.05)	0.74 (0.51, 1.08)	0.75 (0.53, 1.07)	0.1
Model 3	1	0.77 (0.54, 1.09)	0.84 (0.59, 1.22)	0.86 (0.58, 1.27)	0.76 (0.51, 1.13)	0.2
Model 4	1	0.78 (0.55, 1.12)	0.88 (0.60, 1.30)	0.93 (0.60, 1.43)	0.87 (0.53, 1.44)	0.6

Q, quintile. Model 1: unadjusted. Model 2: adjusted for age. Model 3: additionally adjusted for monthly household income, body mass index, educational levels, physical activity levels, residential area, smoking status, alcohol consumption, and hypertension and diabetes at baseline. Model 4: model 3 plus additionally adjusted total energy intake, three major nutrient factors identified in the principal component analysis and use of dietary supplement.

## Supplementary Materials

The following are available online at https://www.mdpi.com/2072-6643/11/7/1484/s1, Figure S1: Flow chart of the participants in the study, KoGES, Korean Genome and Epidemiology study, Table S1: Factor loading matrix for the factor analysis of nutrients.
