# Supplementary material for: Dietary Vitamin B6 Intake Associated with a Decreased Risk of Cardiovascular Disease: A Prospective Cohort Study
Source: Nutrients. 2019 Jun 29;11(7):1484. doi: 10.3390/nu11071484 (PMC6682858; doi:10.3390/nu11071484)
Supplement: Supplementary file 1 [file nutrients-11-01484-s001.zip › Figure S1_╝÷┴ñ║╗(├╓┴╛) (1).pdf]

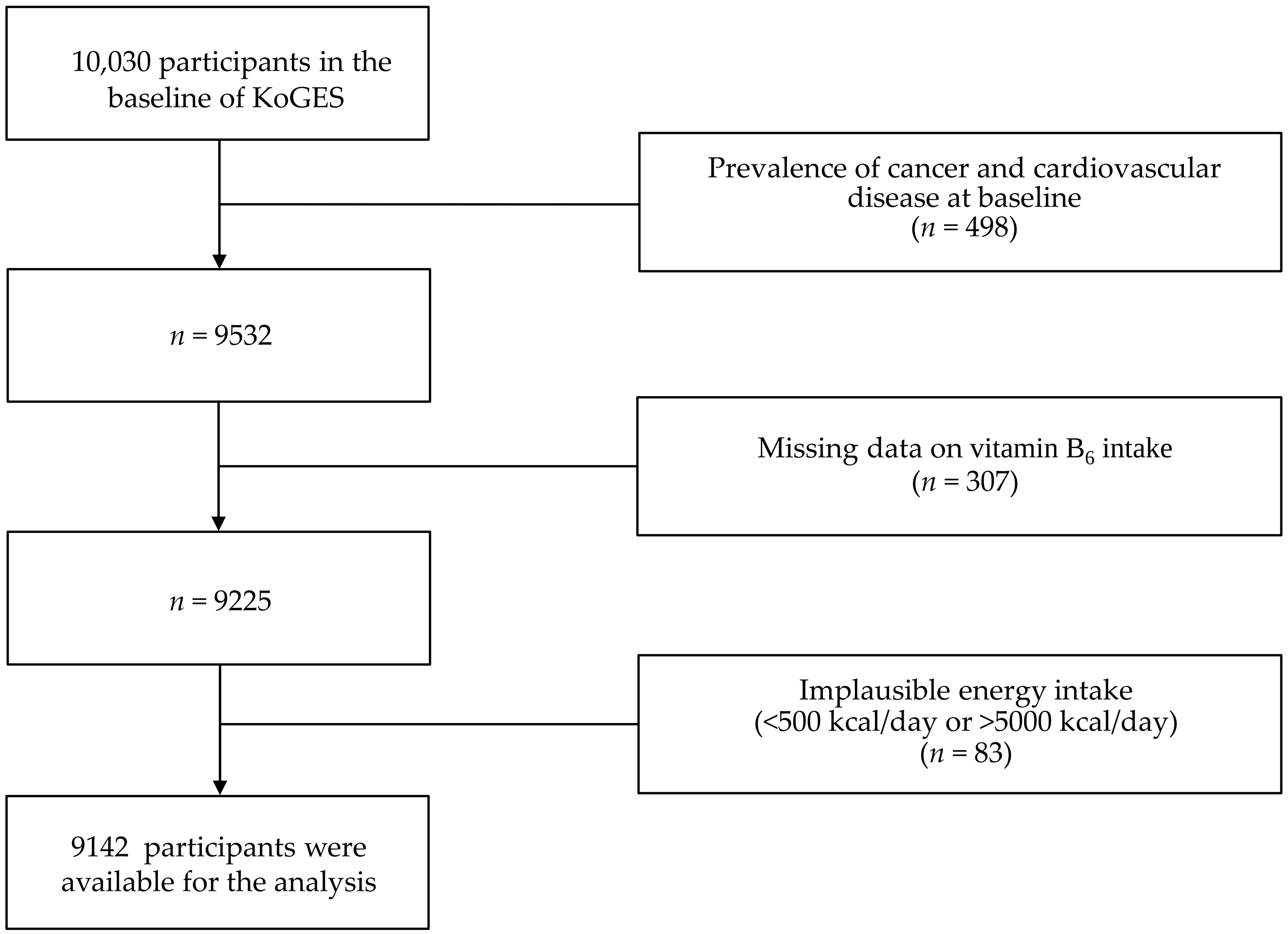

Figure S1. Flow chart of the participants in the study. KoGES, Korean Genome and Epidemiology study.
