# Supplementary material for: Dietary Vitamin B6 Intake Associated with a Decreased Risk of Cardiovascular Disease: A Prospective Cohort Study
Source: Nutrients. 2019 Jun 29;11(7):1484. doi: 10.3390/nu11071484 (PMC6682858; doi:10.3390/nu11071484)
Supplement: Supplementary file 1 [file nutrients-11-01484-s001.zip › Tabls S1_╝÷┴ñ║╗(├╓┴╛) (1).docx]

| **Table S1.** Factor loading matrix for the factor analysis of nutrients. | | | |
| --- | --- | --- | --- |
| Variable | Factor 1 | Factor 2 | Factor 3 |
| Fiber | 93* | 2 | -3 |
| Folate | 92* | 15 | 11 |
| Carotene | 84* | 14 | 6 |
| Vitamin A | 83* | 19 | 20 |
| Potassium | 82* | 30 | 31 |
| Vitamin C | 80* | 4 | 12 |
| Iron | 77* | 38 | 23 |
| Sodium | 74* | 15 | -4 |
| Vitamin E | 64* | 30 | 29 |
| Fat | -7 | 82* | 39 |
| Protein | 30 | 81* | 39 |
| Niacin | 41 | 78* | 7 |
| Thiamin | 49 | 72* | -1 |
| Zinc | 22 | 49 | 20 |
| Carbohydrate | 6 | -90* | -36 |
| Retinol | -1 | 23 | 91* |
| Cholesterol | 3 | 47 | 69* |
| Calcium | 57* | 21 | 67* |
| Riboflavin | 46 | 50 | 63* |
| Phosphorus | 51* | 49 | 56* |
| Values are multiplied by 100 and rounded to the nearest integer.  * Values greater than 0.505143. | | | |
